# The Romberg sign, unilateral vestibulopathy, cerebrovascular risk factors, and long-term mortality in dizzy patients

**DOI:** 10.3389/fneur.2022.945764

**Published:** 2022-08-05

**Authors:** Jan Erik Berge, Frederik Kragerud Goplen, Hans Jørgen Aarstad, Tobias Andre Storhaug, Stein Helge Glad Nordahl

**Affiliations:** ^1^Norwegian National Advisory Unit for Vestibular Disorders, Haukeland University Hospital, Bergen, Norway; ^2^Department of Otorhinolaryngology and Head and Neck Surgery, Haukeland University Hospital, Bergen, Norway; ^3^Department of Clinical Medicine, University of Bergen, Bergen, Norway; ^4^Department of Anesthesiology and Intensive Care, Vestre Viken Hospital Trust, Drammen, Norway

**Keywords:** survival, posturography, dizziness, vertigo, vestibular disorders, caloric response, balance

## Abstract

**Objectives:**

Describe the relationship between unsteadiness, canal paresis, cerebrovascular risk factors, and long-term mortality in patients examined for dizziness of suspected vestibular origin.

**Study design:**

Observational cohort with prospective collection of survival data.

**Setting:**

University clinic neurotological unit.

**Patients:**

Consecutive patients aged 18–75 years examined in the period 1992–2004 for dizziness of suspected vestibular origin.

**Outcome measures:**

Overall survival. Standardized mortality ratio (SMR). Factors: Unsteadiness, canal paresis, age, sex, patient-reported diabetes, hypertension, heart disease, stroke, or TIA/minor stroke. Patients were classified as steady or unsteady based on static posturography at baseline compared to normative values.

**Results:**

The study included 1,561 patients with mean age 48 years and 60 % females. Mean follow-up was 22 years. Unsteadiness was associated with higher age, heart disease, diabetes, hypertension, and cerebrovascular dizziness. There were 336 deaths over 31,335 person-years (SMR 0.96; 95 % confidence interval: 0.86–1.07). Canal paresis was not related to unsteadiness (chi square: *p* = 0.46) or to mortality (unadjusted Cox hazard ratio: 1.04, 95% CI: 0.80–1.34). Unsteadiness was an independent predictor of mortality (adjusted Cox hazard ratio: 1.44, 95% CI: 1.14–1.82).

**Conclusions:**

Unsteadiness measured by static posturography is associated with higher age, known cerebrovascular risk factors, and with increased long-term mortality, but not with canal paresis in patients evaluated for dizziness. The study highlights the importance of evaluating patients with conspicuous postural instability for non-vestibular causes.

## Introduction

The Romberg sign (Moritz Romberg 1795–1873) is present when a patient tends to sway or fall while standing with feet together and eyes closed. It was first described in the 19^th^ century as a useful indicator of proprioceptive loss due to neurosyphilis (tabes dorsalis) (1). Romberg himself was probably unaware that a similar disruption of balance may be caused by vestibular loss (2). This ambiguity complicates the interpretation of the Romberg sign in clinical practice.

In patients with acute vestibular symptoms, commonly seen in emergency departments, oculomotor signs denoted by the acronym “HINTS” are more suitable than the Romberg test to single out patients with a central lesion–for example due to a posterior circulation stroke–as opposed to more common benign peripheral vestibular disorders (3). Marked postural sway may be present in both central and peripheral lesions. However, in patients with severe truncal ataxia who are unable to sit or stand without support, a central lesion is usually suspected (4).

In patients with episodic or chronic vestibular symptoms, commonly seen in outpatient clinics, the spectrum of possible causes is even wider, and even advanced posturographic systems, have been found to have limited diagnostic value (5). If the simple Romberg test is to be used at all in this setting, the clinician would be right to ask whether marked postural instability with eyes closed should be interpreted as a sign of a peripheral vestibular problem, or rather of a proprioceptive or central nervous disease and whether this finding has implications for the prognosis of the patient, for example with respect to long-term survival.

Patients suffering from dizziness or vertigo are often found to have benign disorders of the peripheral vestibular system (6), and severe underlying conditions have, to some extent, been ruled out by referring physicians (7). Nevertheless, a considerable proportion suffers from disorders of unclear or complex etiology, and some may have more serious underlying disorders including cerebrovascular disease. Dizziness and unsteadiness are sometimes indicators of serious disease. In a large population-based study, Corrales et al. (8) found a near doubling of mortality (OR 1.7, 95% CI 1.4–2.2) in adult Americans reporting dizziness or balance problems after adjusting for age, sex, education, ethnicity, race, diabetes, cardiovascular or cerebrovascular disease, and cancer. This study did not differentiate between dizziness and unsteadiness. Other studies have shown that postural instability, quantified by different clinical scoring systems, is associated with increased mortality in elderly (9) and middle-aged persons (10).

In a previous study, we found that standardized mortality ratio in patients examined for dizziness of suspected vestibular origin was the same as in the general population (11). This implies that vestibular symptoms *per se* are not necessarily a sign of serious underlying disease. On the contrary, such symptoms are often caused by benign vestibular disorders, the most common being benign paroxysmal positional vertigo, vestibular migraine, and persistent postural-perceptual dizziness. However, increased mortality was found in a subgroup of patients reporting unsteadiness between dizziness attacks. This suggests that it is of importance to differentiate the symptoms of dizziness/vertigo from that of unsteadiness.

The purpose of this study was to examine the value of an objective measure of standing balance, a version of the Romberg test–static posturography with eyes closed–with respect to its ability to discriminate patients with unilateral vestibulopathy–as measured by the caloric test–from patients with more serious underlying disorders–as measured by comorbidity and long-term mortality.

## Materials and methods

### Patients and setting

This is a study of survival data gathered prospectively from a cohort of consecutive patients aged 18–75 years examined between 1992 and 2004 in a neurotological laboratory at the Department of Otorhinolaryngology and Head and Neck Surgery at Haukeland University Hospital in Bergen. The subjects were mostly outpatients referred for suspected vestibular disorder. For patients seen more than once during the study period, only data from the first examination was included in the study.

### Ethics

The study was approved by the Regional Committee for Medical and Health Research Ethics of Western Norway (2012/1075/REK vest).

### Survival data

Survival data were obtained from the Norwegian National Population Register and updated as per 31 January 2021. Standardized mortality ratios (SMR) were calculated based on life tables by sex and age published by Statistics Norway (12).

### Static posturography

All patients underwent static posturography as described previously (13). Briefly, the patient was asked to stand quietly on a static force platform (Cosmogamma^®^, AC International, Cento, Italy) for 60 s with eyes open and then for 60 s with eyes closed. The visual surroundings were kept constant and the room quiet. The platform contained three strain gauge transducers connected to a computer that calculated the center of pressure (COP) exerted by the patient's feet on the platform while maintaining balance. The length of the curve in millimeters (path length) described by the COP during each examination was used as the main parameter indicating postural instability. The path length may vary from zero–the theoretical result of an immovable object being placed on the platform–to several thousands, indicating severe postural instability. Normative values were taken from a previous study (13). Path lengths >895 millimeters with eyes open or 1,665 millimeters with eyes closed were considered abnormal.

### Caloric testing

All patients underwent bithermal (44 and 30 degrees centigrade) caloric testing after static posturography, and caloric asymmetry was calculated according to Jongkees' formula. Caloric asymmetry >25 % was considered abnormal and defined as a canal paresis.

### Clinical data and covariates

The clinical diagnoses were reviewed retrospectively by two of the co-authors (FG, SHGN). Patients were divided into four age groups (18–39, 40–49, 50–59, and 60–75 years). Cardio-vascular comorbidities were evaluated clinically based on information given by the patient and examining physicians. Included covariates were canal paresis, patient-reported hypertension, diabetes, history of stroke, TIA/minor stroke (TIA, transitory ischemic attack), or heart disease. In addition, some of the patients received a diagnosis of dizziness of suspected cerebrovascular origin. This was a clinical diagnosis made by an otorhinolaryngologist and not based on explicit criteria or imaging. It was rarely indicative of an acute stroke since most patients were seen in an elective, outpatient setting. However, since dizziness of cerebrovascular origin may influence survival, this diagnosis was included among the risk factors in the study.

### Statistical analysis

For statistical analysis and data interpretation, two variables of postural sway were used. First, a continuous variable was made by averaging path length with eyes open and eyes closed. This variable was then stratified by quartiles to four levels indicating low, low-median, median-high, or high postural instability. Second, a dichotomized variable was created using previously published normative data (13). If the path length was outside normal limits either with eyes open or closed, the patient was characterized as “unsteady.” Otherwise, the patient was considered “steady.”

Cox proportional hazards regressions models were used to calculate crude and adjusted hazard ratios for survival predicted by age, sex, postural instability, diabetes, hypertension, heart disease, stroke, TIA/minor stroke, and dizziness of cerebrovascular origin. Adjusted hazard ratios were reported after backward stepwise elimination of non-significant factors. Follow-up time was defined as the time interval between the first examination and the last update of survival data (31 January 2021).

Statistical analysis was performed using R 4.0.3 (R Foundation for Statistical Computing, Vienna, Austria), the Epi (14) and popEpi packages (15). Two-sided *p*-values < 0.05 were considered significant.

## Results

**Figure 1:**
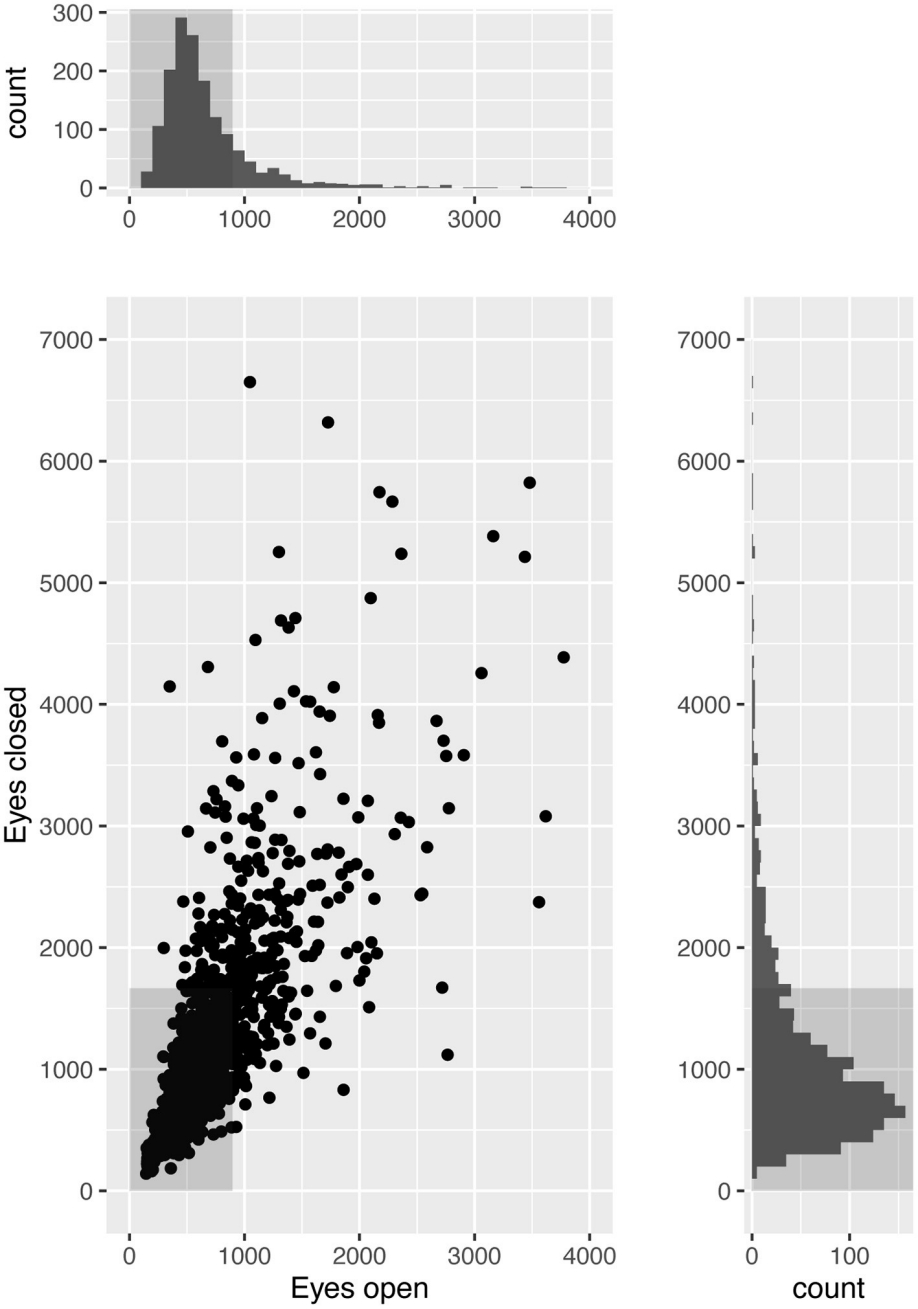
Static posturography results in 1,561 patients examined for dizziness of suspected vestibular origin. Scatterplot with marginal histograms showing postural sway while standing quietly on a static force platform for 60 s with eyes open and closed. Plotted values are the length in millimeters of the path described by the center of pressure under the patient's feet. Gray boxes indicate normal limits.

**Figure 2:**
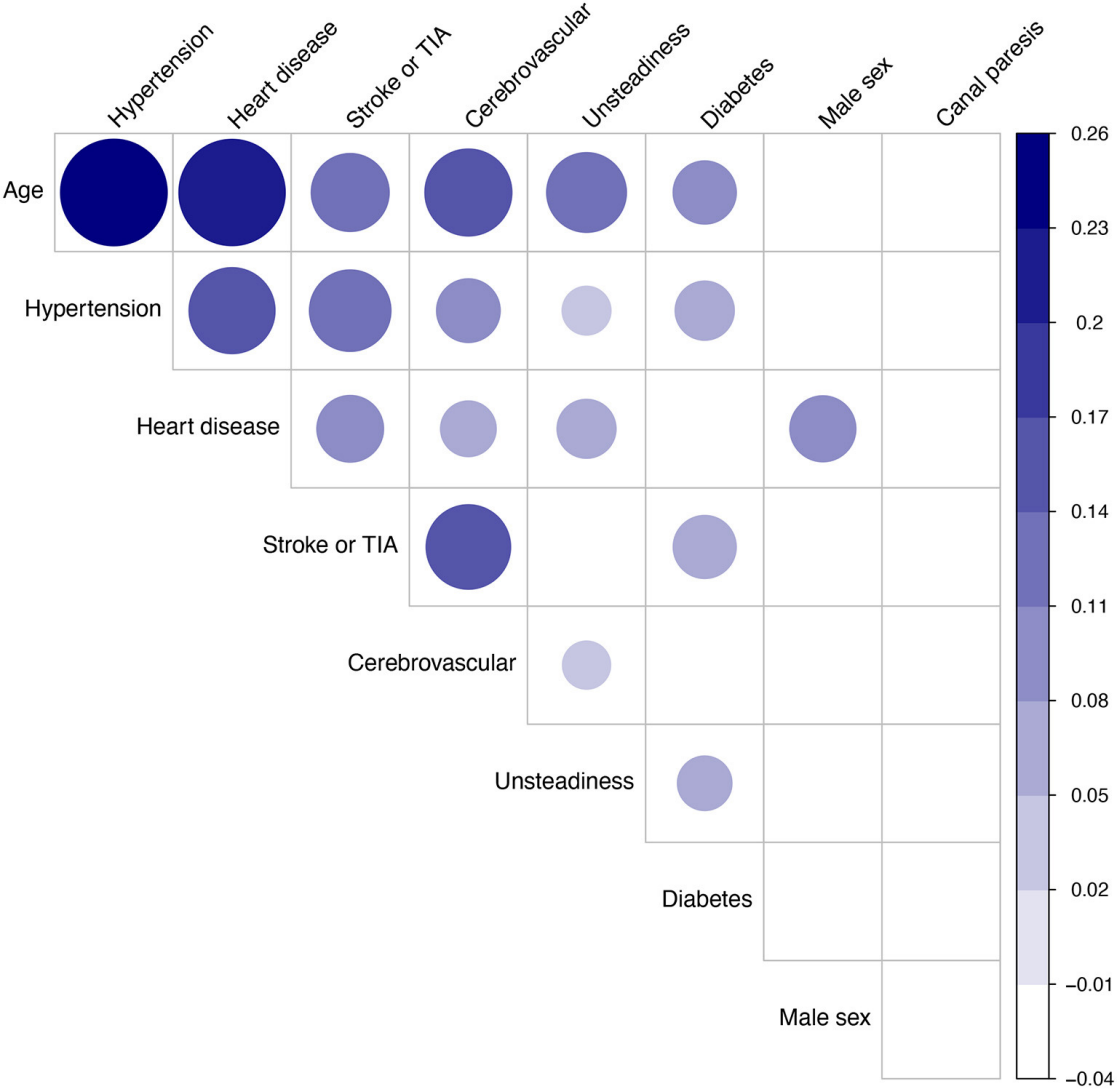
Correlation matrix analysis of nine candidate factors, prior to survival analysis, in 1,561 patients examined for dizziness of suspected vestibular origin. Dots indicate significant correlations (*p* < 0.05). Dot size and darkness indicate strength of correlation (Pearson's R). Factor ordering: first principal component order.

Out of 1,796 patients with complete data on posturography and clinical covariates, 84 patients were excluded due to missing consent or unknown vital status, and further 151 due to being outside the age-range 18–75 years at baseline. Descriptive data for the remaining 1,561 participants are presented in Table 1 with clinical diagnoses in Table 2. Results from the caloric test were available for 1,326 patients of which 28.5% had a canal paresis. Dizziness of suspected cerebrovascular origin was noted in 106 (6.8%) of the patients. Posturography results are shown in Figure 1 and the correlation matrix between risk factors is shown in Figure 2.

**Table 1:** Descriptive data of participants (*n* = 1,561).

**Parameter**	**Values**
Age (years); *mean, SD*	48.4, 14.0
18–39 years; *n*	444
40–49 years; *n*	369
50–59 years; *n*	391
60–75 years; *n*	357
Female; *n, %*	934, 59.8
**Posturography**	
Unsteady patients; *n, %*	357, 22.9
Path length (mm)^*^; *quartiles (25%, 50%, 75%)*	521, 724, 1,069
Caloric test; *n*	1,326
Canal paresis; *n, %*	378, 28.5
**Patient-reported comorbidities**	
Diabetes; *n, %*	30, 1.9
Hypertension; *n, %*	213, 13.6
Heart disease; *n, %*	84, 5.4
Stroke or TIA; *n, %*	26, 1.7
**10-year survival (percent)**	
*Observed, lower CI, upper CI*	93.9, 92.7, 95.0

TIA, transitory ischemic attack; n, count; SD, standard deviation; mm, millimeters; CI, 95 % confidence interval.

* Arithmetic mean of path length with eyes open and eyes closed.

**Table 2:** Clinical diagnoses in 1,561 patients examined in a university clinic for suspected vestibular disorder.

**Diagnosis**	**Count**	**Percent**
**Peripheral vestibular**
Benign paroxysmal positional vertigo	209	13.4 %
Vestibular neuritis	184	11.8 %
Labyrinthitis	26	1.7 %
Menière's disease	175	11.2 %
Vestibular schwannoma	63	4.0 %
**Other ear and hearing disorders**
Otosclerosis	8	0.5 %
Sudden deafness	8	0.5 %
Chronic otitis media	1	0.1 %
Other middle ear disease	11	0.7 %
Hearing loss NOS	15	1.0 %
**Trauma**
Perilymphatic fistula	7	0.4 %
Skull fracture	13	0.8 %
Head injury without fracture	24	1.5 %
Whiplash	11	0.7 %
Decompression sickness	6	0.4 %
Barotrauma	4	0.3 %
**Neurological disorders**
Cerebrovascular	106	6.8 %
Central vestibular NOS^*^	148	9.5 %
Multiple sclerosis	6	0.4 %
Borreliosis	1	0.1 %
Epilepsy	3	0.2 %
**Other**
Drug induced	4	0.3 %
ME	1	0.1 %
Postinfectious	29	1.9 %
Cervicogenic	136	8.7 %
Congenital	2	0.1 %
Psychogenic	15	1.0 %
Non-otogenic NOS	274	17.6 %

NOS, not otherwise specified; ME, myalgic encephalopathy; CNS, central nervous system.

* Including vestibular migraine.

Follow-up time ranged from 17 to 29 years (mean 22, SD 2.9 years). The observed number of deaths in the study population was 336 over a total of 31,335 person-years, which did not differ significantly from the expected number of 350 deaths in the Norwegian general population matched for age, sex, and calendar years (standardized mortality ratio: 0.96, 95 % confidence interval: 0.86–1.07).

Results of the Cox regression analysis are shown in Table 3. Unsteadiness on static posturography was a significant predictor of mortality independent on age, sex, self-reported comorbidities, and clinical diagnosis of dizziness of suspected cerebrovascular origin with an adjusted hazard ratio of 1.438 (95% CI: 1.138–1.815). Self-reported diabetes and stroke or TIA/minor stroke were also significant predictors in the adjusted analysis.

**Table 3:** Cox regression analysis of long-term survival in 1,561 patients examined for dizziness of suspected vestibular origin.

	**Univariate**				**Adjusted**			
	**95% CI**				**95% CI**			
**Factor**	**HR**	**lower**	**upper**	***p***	**HR**	**lower**	**upper**	***p***
**Age**								
18–39 yr	reference				reference			
40–49 yr	5.878	2.605	13.26	<0.0001	5.685	2.519	12.828	<0.0001
50–59 yr	12.548	5.769	27.29	<0.0001	11.416	5.244	24.854	<0.0001
60–75 yr	65.342	30.765	138.78	<0.0001	59.857	28.141	127.322	<0.0001
**Sex**								
Male	1.384	1.117	1.714	0.00298	1.379	1.111	1.711	0.00350
**Self-reported comorbidity**								
Diabetes	4.162	2.618	6.618	<0.0001	2.089	1.307	3.340	0.00207
Hypertension	2.630	2.059	3.358	<0.0001				
Heart disease	4.339	3.210	5.865	<0.0001				
Stroke or TIA/minor stroke	4.615	2.869	7.424	<0.0001	2.034	1.256	3.295	0.00392
**Clinical diagnosis**								
**Dizziness of cerebrovascular origin**	2.179	1.566	3.032	<0.0001				
**Static posturography**								
Unsteady^*^	1.831	1.453	2.306	<0.0001	1.438	1.138	1.815	0.00229

Univariate and adjusted hazard ratios after backward stepwise elimination of non-significant factors.

CI, confidence interval; HR, hazard ratio.

* Path length outside normative values with eyes open or closed.

**Figure 3:**
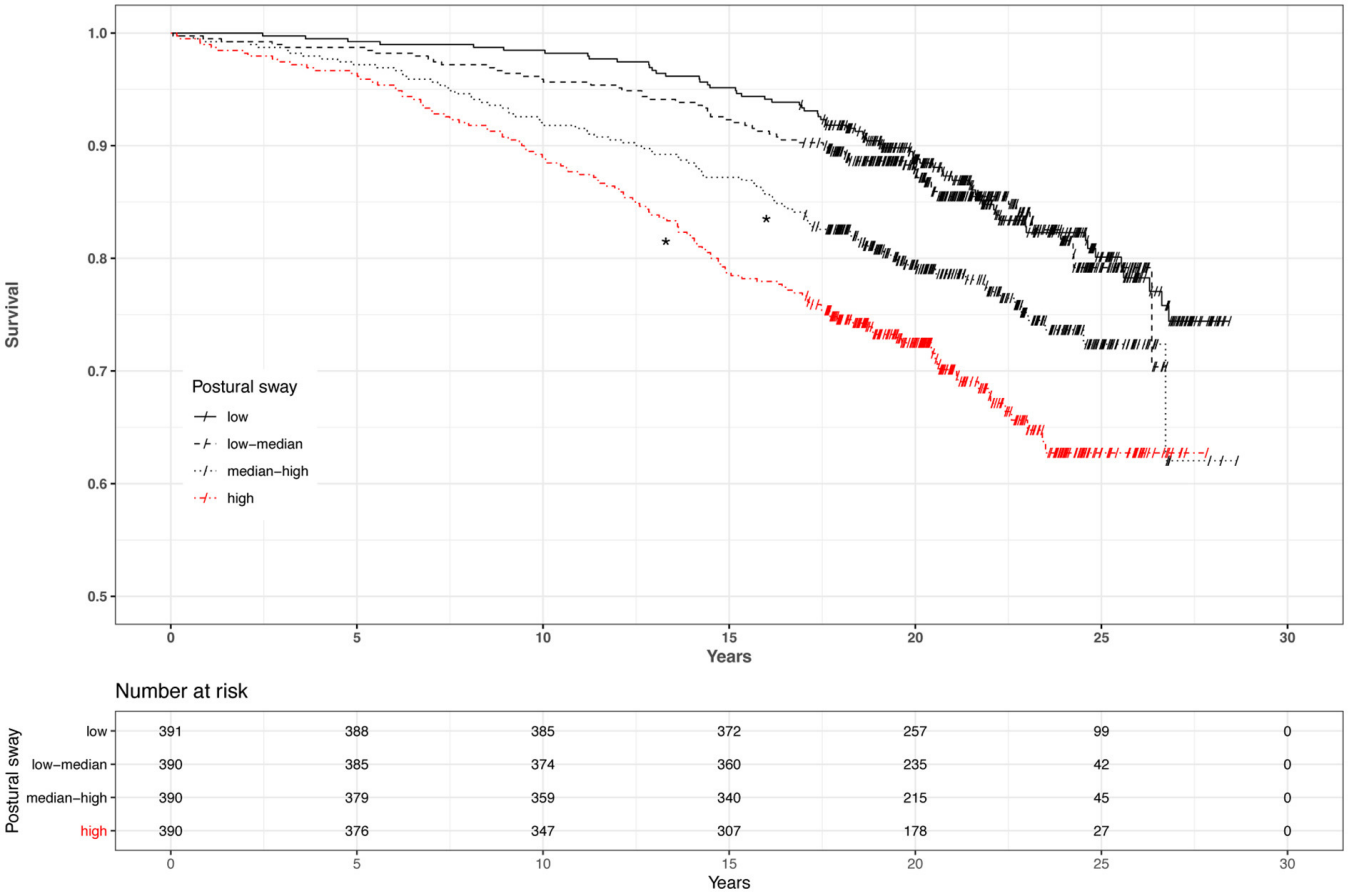
Kaplan-Meier survival in 1,561 dizzy patients and postural instability stratified by quartiles. Postural sway measured while standing quietly on a force platform for 60 s with eyes open and closed. Movement of the center of pressure was measured in millimeters, and results with eyes open and closed were averaged. Censoring events are marked with a slash (/). *Significant (Cox regression, *p* < 0.005).

Kaplan-Meier analysis of survival related to four increasing levels of postural instability is shown in Figure 3. The two groups with median-high or high postural sway had decreased survival compared to the group with low postural sway (Cox regression, *p* < 0.005).

The presence of a canal paresis (caloric asymmetry >25 %) was not associated with postural instability (chi square: *p* = 0.44), nor with mortality (HR 1.036, 95% CI: 0.8002–1.34).

## Discussion

In this cohort of patients examined for dizziness of suspected vestibular origin, postural instability was not associated with unilateral vestibulopathy, but rather with increasing age, cerebrovascular risk factors and increased long-term mortality. Unsurprisingly, a canal paresis was not associated with mortality. To the best of our knowledge, this is the first study to show the relationship between an objective measure of postural sway and long-term survival in dizzy patients. Combined with the patient-reported comorbidities, a relatively simple measure of postural instability may provide prognostic information in patients undergoing evaluation for dizziness. This finding underscores the importance of evaluating unsteadiness, and of differentiating this from dizziness and vertigo, in patients with vestibular symptoms.

Cerebrovascular risk factors contributed significantly to mortality, which was expected since stroke is one of the leading causes of death in Europe (16). In the acute setting, dizziness and vertigo are sometimes caused by a posterior circulation stroke (17), and even when this has been excluded, a study has indicated increased risk of a cerebrovascular event after hospital discharge (18). Moreover, a recent study provides evidence that transient isolated vertigo or dizziness may sometimes be symptoms of TIA (19). However, the present study was performed in an elective setting, and patients with suspected cerebrovascular cause of their symptoms had presumably been screened out, to some extent, by referring physicians. The diagnosis of dizziness of suspected cerebrovascular origin did not contribute to the prognosis after adjustment for patient-reported comorbidities. The reason for this may be that the major risk factors of stroke–i.e., age and patient-reported diabetes, hypertension, atrial fibrillation, and previous stroke or TIA/minor stroke–were accounted for in the adjusted analysis. Hence, the clinical diagnosis of cerebrovascular dizziness without additional objective information, such as biochemical markers, MRI or Doppler imaging findings, did not provide additional prognostic value. It is nevertheless interesting that posturography remained a significant predictor even after correction for these factors.

Previous studies have documented mortality in patients suffering from vestibular symptoms (8, 9, 20). In a large population-based study, Corrales et al. (8) found a near doubling of mortality (OR 1.7, 95% CI 1.4–2.2) in adult Americans reporting dizziness or balance problems after adjusting for age, sex, education, ethnicity, race, diabetes, cardiovascular or cerebrovascular disease, and cancer. In our study of dizzy patients, the standardized mortality rate was the same as in the general population. This may be explained by patient selection and screening by referring physicians, since the main purpose of the examination was to uncover vestibular disorders. The benign nature of these disorders is supported by our finding that caloric asymmetry was not associated with increased mortality. Van Vugt et al. (20) found a 40.5% 10-year mortality in a group of elderly patients with dizziness in primary care. This was not compared to standardized mortality rates. Moreover, the patients had a mean age of 79 years at inclusion, which is considerably higher than in our study. The finding by van Vugt et al. (20) that patients with vertigo had lower mortality than those with dizziness of other types is interesting, and agrees with a previous study from our group (7). In the latter study, patient-reported unsteadiness between dizziness episodes was associated with higher mortality.

Other studies have found that gait and balance problems can be used to predict mortality. Blain et al. (21) studied a population of community-dwelling women aged ≥75 years and found that 8-year survival was related to balance and walking speed after adjusting for a wide range of covariates. Cooper et al. (10) found that all-cause mortality in a group of 2,766 53-year-olds was related to measures of physical capability, specifically grip strength, chair rise speed and standing balance time. The authors found some evidence that the timed one-leg stance test with eyes closed was the factor most strongly associated with survival. A linear relationship between this test and path length from posturography has been reported in a previous study (22) indicating a partial overlap between these two methods.

A similar-sized study from Finland reported no association between posturographic unsteadiness and long-term mortality in a population-based cohort of 1,568 women (23). A positive association was found in the crude analysis between mortality and anteroposterior, mediolateral and total maximum amplitude of the COP. However, the association was lost after adjustment for age, parents' hip fracture, smoking and leg-extension strength. The study also found postural sway to be associated with osteoporotic fractures. Possible explanations for the difference in outcomes may include different sway parameters (maximum sway amplitude vs. total path length), test conditions (eyes open vs. average between eyes open and closed) and patient selection (population sample of women vs. dizzy patients of both sexes). However, since the crude analysis revealed similar results in the two studies, the difference may also be explained by the difference in covariates. The authors of the Finnish study suggested that the association between postural sway and mortality is indirect, and that “sway is more of an indicator for general health status.” This is in line with our study, finding sway to be mostly correlated with age and other cerebrovascular risk factors.

The lack of association between postural sway and caloric asymmetry is not surprising (11). Most of the patients were seen in an elective setting due to chronic or episodic symptoms. While a vestibular lesion leading to canal paresis typically causes marked unsteadiness in the acute phase, the symptoms tend to improve due to central vestibular compensation. Thus, a disease leading to asymmetric caloric function, for example a sequela to vestibular neuritis or vestibular schwannoma, may sometimes lead to less postural unsteadiness than an episodic disorder with symmetric caloric response, like BPPV or Menière's disease, or a chronic neurological or orthopedic problem.

Apart from underlying cardio- and cerebrovascular disorders, possible causal relationships between unsteadiness and mortality could involve general frailty and risk of falling. In a study of relatively active home-dwelling elderly persons with mean age 70 years, Tuunainen et al. (24) concluded that vertigo and poor postural stability constituted the major reasons for falling. Falls are a major cause of morbidity in the elderly population (25, 26), and even though vertigo of peripheral vestibular origin may not be associated with increased all-cause mortality–as our study indicates–falls rank among the leading causes of death (26). Part of the excess mortality in patients with postural instability might therefore be explained by falls.

Strengths of the present study include the objective measuring of postural balance and caloric asymmetry, the long follow-up (mean 22 years) and wide age range (18–75 years) in a large population of dizzy patients examined for suspected vestibular disorder. The long follow-up and inclusion of relatively young patients compared to previous studies, means that the study has potential for early detection and preventive measures related to long-term survival. The risk of attrition bias was considered by the authors and found to be low since <5 % of the cohort was lost to follow-up due to missing consent or unknown vital status at follow-up. The addition of standardized mortality ratios gives valuable information about the study sample in comparison to the general population.

Limitations include the fact that the patients were seen in a specialized clinic in an elective outpatient setting. The results are not necessarily applicable to patients seen for acute vestibular syndrome. Other studies indicate that people suffering from dizziness in the general population has a higher overall mortality (8). However, this does not invalidate the association between mortality and postural instability found in the present study, which agrees with other studies of less selected populations (10, 21). Since the causes of death were unknown, the direct causal link between postural instability and mortality cannot be ascertained. Future studies on disease specific mortality in patients with postural instability are needed. Until further research is available, dizzy patients with conspicuous unsteadiness should be evaluated for cerebrovascular risk factors. Preventive measures should focus on minimizing the risk of stroke and falls.

In conclusion, this study found that the Romberg sign in patients undergoing elective evaluation for vestibular symptoms was related to age and cerebrovascular risk factors including hypertension and diabetes as well as being an early predictor of mortality. It was not related to unilateral vestibulopathy as measured by the caloric test. This finding underscores the importance of differentiating objective unsteadiness from the subjective feeling of vertigo or dizziness. Patients with conspicuous unsteadiness with eyes closed require diagnostic evaluation for non-vestibular etiology and fall-risk.

## Data availability statement

The original datasets presented in this article are not readily available due to Norwegian data protection legislation. Requests to access aggregated data should be directed to Frederik Kragerud Goplen (frederik.kragerud.goplen@helse-bergen.no).

## Ethics statement

The study was approved in advance by the Regional Committee for Medical and Health Research Ethics of Western Norway (2012/1075/REK vest). Active consent was not required according to Norwegian legislation. Living participants were informed in writing about the study and given the opportunity to withdraw by phone, e-mail or by returning a prepaid envelope.

## Author contributions

JB, FG, and TS: data collection. JB and FG: data analysis and drafting the manuscript. All authors contributed in concept and design of the study, revision of the article for important intellectual content, and approval of the submitted version.

## Conflict of interest

The authors declare that the research was conducted in the absence of any commercial or financial relationships that could be construed as a potential conflict of interest.

## Publisher's note

All claims expressed in this article are solely those of the authors and do not necessarily represent those of their affiliated organizations, or those of the publisher, the editors and the reviewers. Any product that may be evaluated in this article, or claim that may be made by its manufacturer, is not guaranteed or endorsed by the publisher.
